# Supplementary material for: Micro-scale functional modules in the human temporal lobe
Source: Nat Commun. 2022 Oct 21;13:6263. doi: 10.1038/s41467-022-34018-w (PMC9587217; doi:10.1038/s41467-022-34018-w)
Supplement: Supplementary file 1 — Supplementary Information [file 41467_2022_34018_MOESM1_ESM.pdf]

Supplementary Methods

Pair-wise conditional Granger causality

In order to estimate directed functional connectivity in local cortical networks we compute the pair-wise conditional Granger causality between all pairs of channels in each MEA in every experimental session. This methodology has been well described in the literature[1–5], and we only provide a brief summary of the method here. We performed all Granger causal calculations and relevant statistics using the Multivariate Granger Causality Toolbox (MVGC)[5]. Intuitively, a variable $Y(t)$ is said to Granger cause variable $X(t)$ if we are better able to predict the future of $X(t)$ by using the past of $Y(t)$ compared to using only the past of $X(t)$. These notions of prediction and precedence in Granger causal analysis are based on vector autoregressive (VAR) modeling. Specifically, one considers the full VAR model,

$$X_t = \sum_{k=1}^{p} A_{xx,k} \cdot X_{t-k} + \sum_{k=1}^{p} A_{xy,k} \cdot Y_{t-k} + \varepsilon_{x,t} \tag{1}$$

which uses the past of both X and Y to predict the future of X, and the reduced VAR model,

$$X_t = \sum_{k=1}^{p} A'_{xx,k} \cdot X_{t-k} + \varepsilon'_{x,t} \tag{2}$$

which only uses the past of X. Here $\varepsilon_{x,t}$ and $\varepsilon'_{x,t}$ are the residuals of the full and reduced models respectively. In this framework, if the variability of the residuals in the full model is significantly less than that of the reduced model then the inclusion of Y improves the prediction of X, and statistical significance can be assessed by way of the $\mathcal{F}$-statistic:

$$\mathcal{F}_{y\to x} \doteq \ln \frac{\mathrm{var}(\varepsilon'_{x,t})}{\mathrm{var}(\varepsilon_{x,t})} \tag{3}$$

A potential confound of using this unconditioned statistic to assess functional connectivity is that spurious causalities can be reported between two variables due to common lagged dependencies on a third variable. These spurious connections can be mitigated by conditioning out the common dependencies, in which case the VAR models become,

$$X_t = \sum_{k=1}^{p} A_{xx,k} \cdot X_{t-k} + \sum_{k=1}^{p} A_{xy,k} \cdot Y_{t-k} + \sum_{k=1}^{p} A_{xz,k} \cdot Z_{t-k} + \varepsilon_{x,t} \tag{4}$$

$$X_t = \sum_{k=1}^{p} A'_{xx,k} \cdot X_{t-k} + \sum_{k=1}^{p} A'_{xz,k} \cdot Z_{t-k} + \varepsilon'_{x,t} \tag{5}$$

with the corresponding conditional $\mathcal{F}$-statistic,

$$\mathcal{F}_{y\to x|z} \doteq \ln \frac{\mathrm{var}(\varepsilon'_{x,t})}{\mathrm{var}(\varepsilon_{x,t})} \tag{6}$$

In a multivariate setting one can condition not just on a single variable Z, but on all other known variables besides X and Y. This defines the pair-wise conditional Granger causality, $\mathcal{F}_{y\to x\,|\,[xy]}$ where $[xy]$ denotes conditioning on all variables besides x and y. For simplicity, we use $\mathcal{F}$ or $\mathcal{F}_{y\to x}$ to denote the conditional case unless otherwise stated.

From each experimental session we extracted 20 blocks, 5 seconds in duration each, for functional connectivity analysis. We treat the 20 blocks from each session as trials and estimate a single VAR model for that session[1]. In this way, we construct a single weighted-directed adjacency matrix for each MEA and session. We estimated the model order for each MEA separately by first computing the Bayesian information criterion of the full model for model orders between $p = 1$ and $p = 30$ for each session. The model order for each individual MEA was very similar for all sessions. Therefore, for each MEA, we used the same model order (the mode) for every experimental session (typically $p \approx 5$, see Supplementary Figure 10).

Supplementary Note 1

Comparison with other measures of functional connectivity

Granger causal methods have been used extensively in the literature to capture directed influences between cortical regions[3,6]. Furthermore, compared to other measures of functional connectivity GC has a higher correspondence to anatomic connectivity and helps reduce the effects of instantaneous field effects[7,8]. Nevertheless, given the prevalence of correlation and coherence based metrics, we computed the following for all electrode pairs: (i) raw correlation coefficient at zero lag, r, (ii) the correlogram estimate of the absolute maximum cross-correlation, $|r(\tau^*)|$, where τ^* is the lag with maximum correlation (correlograms constructed using 1 second windows with 50% overlap for delays from -250 to 250ms), and (iii) the magnitude squared coherence spectrum, $C(f)$, and maximum coherence, $C(f^*)$, where f^* denotes the frequency of maximum coherence. Power spectral densities for frequencies from 0 to 100Hz (1Hz steps) were estimated using Welch's method with 1 second windows and 50% overlap. We then used the modules from the main results (Granger causality based) to separate these metrics into within-module and across-module pairs.

In individual sessions, correlations between within-module pairs are positive and of large magnitude, whereas correlations between across-module pairs are negative and of small magnitude (Supplementary Figure 3a). Within-module correlations were significantly higher than across module for all individual arrays as well as across all arrays (Supplementary Figure 3b). This clear separation in correlations between within and across module pairs was also evident in individual sessions and for most arrays when using a correlogram estimate of the absolute maximum cross-correlation (Supplementary Figure 3c,d). Lastly, similar results hold when analyzing both the coherence spectrum as well as the maximum coherence. Supplementary Figure 3e shows that for all sessions (thick lines) the average coherence for all frequencies is larger for within-module pairs when compared to across-module pairs. Extracting the maximum coherence for each pair we find that this separation holds for most arrays and is significant across arrays (Supplementary Figure 3f). In summary, while there could be quantitative differences in the identified modules depending on what functional connectivity metric is used, the metrics considered here are capturing similar information, and the main defining feature of modularity (strong within-module connectivity vs weak across-module connectivity) will hold independent of the exact measure of functional connectivity that is used.

Supplementary Note 2

Effect of resolution and between-layer coupling parameters

To quantify the effects of varying the resolution parameter γ and the between-layer coupling parameter ω, we repeat the modularity maximization procedure while varying γ from 0 to 2 in steps of 0.2 and varying ω between 0 and the maximum within-layer connection weight in the network. We use a non-uniform grid of ω values to better estimate the dependencies for both small and large values of ω. We chose to analyze partition quality, persistence, and module diameter as functions of γ for $\omega = \omega^*$ and as functions of ω for

$\gamma = \gamma^* = 1$ to assess the dependence on these parameters, where (γ^*, ω^*) denote the parameters used in the main paper (see Supplementary Figure 6 for an example MEA).

The between-layer coupling parameter, ω, can be thought of as the cost of breaking the within-layer community structure in favor of increasing temporal persistence. This parameter cannot be too small, since this corresponds to treating the layers independently, or too large, which forces all nodes to remain in the same community across layers. The persistence as a function of ω for $\gamma = \gamma^*$ is zero for $\omega = 0$, since there is no coupling between the different layers, and jumps to a finite non-zero value for $\omega > 0$. As ω increases, nodes from different layers are merged, and the persistence increases and eventually plateaus at 1 for both real and surrogate networks. In the main paper we set ω^* to be the median of the non-zero connection weights, which is within the region where the persistence is neither 0 nor 1. Throughout this region, the persistence of the real networks is larger than for the surrogate networks. The results of simulations for all MEAs are qualitatively the same as for the example MEA shown in Supplementary Figure 6

The parameter γ is known as the resolution parameter, and controls the size of the modules discovered. As $\gamma \to 0$ for $\omega = \omega^*$ the quality and persistence of both real and surrogate networks approach 1, and the module diameter approaches the size of the entire array (Supplementary Figure 6). This is expected since the optimal partitioning in this case is just to place all nodes in a single module. As γ increases, the module diameter for the real networks decreases sharply around $\gamma = 0.3 - 0.4$, however, for a wide range of γ values ($\approx$ 0.8-2) the average module size remains in the range of $\approx$ 1-1.5mm. For this range of γ values the quality and persistence of the real networks change slowly and are larger than for the surrogate networks. The results of simulations for all MEAs are qualitatively the same as for the example MEA shown in Supplementary Figure 6. This multi-resolution analysis demonstrates that there is a characteristic size to the identified modules independent of the exact value of the resolution parameter.

We also leveraged the fact that we have arrays of different sizes to further strengthen our claims about the size of the detected modules. In Supplementary Figure 5c we show the module diameter and spatial compactness for the 4mm $\times$ 4mm and 3.2mm $\times$ 3.2mm arrays. In general, there does not appear to be a clear systematic difference between the two array types. The diameter for A2 (4$\times$4mm) is about the same (and sometimes smaller) than for the 3.2$\times$3.2mm arrays. The modules for A1 (4$\times$4mm) do have the largest diameter, however, this appears to be driven by the fact that the modules in this array are the among the least compact. We can see that the MEA with the second largest module diameter is A3 which is 3.2$\times$3.2mm, and this array also has the second smallest compactness. Hence, the differences in module diameter tend to arise from differences in compactness rather than differences in array size.

Supplementary Note 3

Within vs across MEA controls on functional connectivity and modularity

For participants with two MEAs we can compute the pair-wise conditional Granger causality using activity from both arrays (Supplementary Figure 11a). As expected given the distance dependent properties of synaptic connectivity, almost all of the connections are constrained to occur between electrodes from the same MEA, with very few, if any, connections between electrodes from different MEAs. This effect was consistent across all participants as demonstrated by a substantially and significantly higher connection probability within array compared to across arrays (.26 $\pm$ .04 for within vs .007 $\pm$ .004 for across; Supplementary Figure 11b). Since the activity between the two MEAs from a single participant is largely independent, modularity optimization on the combined network produces modules that are constrained to consist of electrodes from a single MEA (Supplementary Figure 11c for an example). For this reason we treated each MEA independently for the functional connectivity analyses in the main paper.

Supplementary Note 4

Contributions to differential module coding

We compared the classification accuracy of individual modules after shuffling neuron identities within a module to the accuracy after shuffling identities with neurons from a different module. The classification accuracy was significantly higher after shuffling within compared to shuffling across modules, hence, category information is more similar when two neurons belong to the same module and more independent between neurons from different modules. Two factors likely contribute to this phenomenon: First, consider a module with high classification accuracy and a second one for which classification is not different from chance; shuffling neuron identities between these modules will lead to a decrease in classification because neurons in the second module are likely not coding for category. Second, when shuffling between modules both with significant classification there is still a drop in classification accuracy that is significant across all modules from all MEAs and sessions ($p < .001$, $t(93) = 8.54$), indicating that even when two modules are tuned to category, they can encode different features. There were 4 sessions with multiple modules within the same MEA that had significant classification, and the module-averaged shuffled within accuracy was higher than the shuffled across accuracy for 3 of the 4 sessions, although the effect was not statistically significant.

Supplementary Tables

	Modularity, Q_{ml}					Stationarity					Persistence					
	μ	σ	μ_{null}	σ_{null}	tstat	μ	σ	μ_{null}	σ_{null}	tstat	μ	σ	μ_{null}	σ_{null}	tstat	df
A1	0.48	0.0027	0.39	0.0095	200	0.83	0.047	0.35	0.032	190	0.91	0.021	0.52	0.026	260	499
A2	0.5	0.0028	0.24	0.0028	1400	0.71	0.092	0.14	0.019	140	0.87	0.043	0.28	0.022	270	499
A3	0.47	0.0033	0.29	0.0093	390	0.58	0.11	0.21	0.047	74	0.73	0.083	0.36	0.058	85	499
A4	0.48	0.0027	0.25	0.0074	640	0.63	0.12	0.18	0.039	81	0.73	0.1	0.32	0.051	81	499
A5	0.51	0.0034	0.32	0.0065	600	0.7	0.082	0.18	0.03	140	0.84	0.051	0.34	0.034	180	499
A6	0.5	0.0034	0.32	0.0053	670	0.59	0.11	0.14	0.023	92	0.78	0.061	0.29	0.027	160	499
A7	0.48	0.0037	0.31	0.0066	520	0.52	0.091	0.19	0.032	79	0.73	0.064	0.35	0.035	120	499
A8	0.43	0.0032	0.23	0.0053	690	0.6	0.074	0.21	0.035	110	0.78	0.047	0.39	0.035	150	499
A9	0.39	0.003	0.21	0.0035	830	0.75	0.11	0.17	0.028	110	0.87	0.052	0.35	0.031	190	499
A10	0.39	0.0031	0.22	0.0041	740	0.69	0.096	0.19	0.032	110	0.83	0.041	0.38	0.031	200	499
A11	0.55	0.0064	0.33	0.0074	500	0.8	0.11	0.18	0.034	120	0.88	0.069	0.34	0.041	150	499
A12	0.39	0.0046	0.2	0.005	610	0.86	0.099	0.23	0.043	130	0.92	0.055	0.39	0.048	170	499
A13	0.42	0.0049	0.25	0.0069	420	0.6	0.11	0.23	0.047	70	0.77	0.079	0.39	0.059	88	499
A14	0.35	0.0048	0.17	0.005	580	0.52	0.11	0.28	0.056	46	0.72	0.1	0.45	0.065	53	499
Across Arrays	0.45	0.058	0.27	0.06	18	0.67	0.11	0.2	0.055	15	0.81	0.071	0.37	0.061	19	13

Supplementary Table 1: Statistics for modularity, stationarity, and persistence
Note: for all t-tests $p < .001$

	Diameter(mm)					Compactness					
	μ	σ	μ_{null}	σ_{null}	tstat	μ	σ	μ_{null}	σ_{null}	tstat	df
A1	1.8	0.12	2.8	0.11	-140	0.7	0.034	0.25	0.016	260	499
A2	1.4	0.049	2.9	0.048	-510	0.95	0.013	0.25	0.015	790	499
A3	1.7	0.092	2.2	0.098	-97	0.68	0.043	0.36	0.031	140	499
A4	1.5	0.093	2.3	0.067	-170	0.78	0.03	0.34	0.027	250	499
A5	0.97	0.061	2.3	0.079	-280	0.95	0.024	0.36	0.027	370	499
A6	1.1	0.051	2.4	0.055	-390	0.97	0.013	0.32	0.023	560	499
A7	1.1	0.051	2.2	0.068	-290	0.93	0.019	0.4	0.028	340	499
A8	1.3	0.047	2.3	0.073	-250	0.92	0.018	0.37	0.026	390	499
A9	1.2	0.023	2.3	0.053	-430	0.94	0.016	0.36	0.024	460	499
A10	1.2	0.041	2.2	0.058	-300	0.94	0.016	0.38	0.025	430	499
A11	1.1	0.038	2.3	0.067	-330	0.98	0.0068	0.37	0.03	440	499
A12	1.1	0.11	2.2	0.069	-190	0.99	0.013	0.41	0.028	430	499
A13	1.3	0.13	2.3	0.083	-150	0.91	0.027	0.35	0.03	310	499
A14	1.5	0.076	2.4	0.099	-160	0.8	0.044	0.36	0.028	200	499
Across Arrays	1.3	0.24	2.4	0.22	-17	0.89	0.1	0.35	0.1	21	13

Supplementary Table 2: Statistics for diameter and compactness
Note: for all t-tests $p < .001$

Supplementary Figures

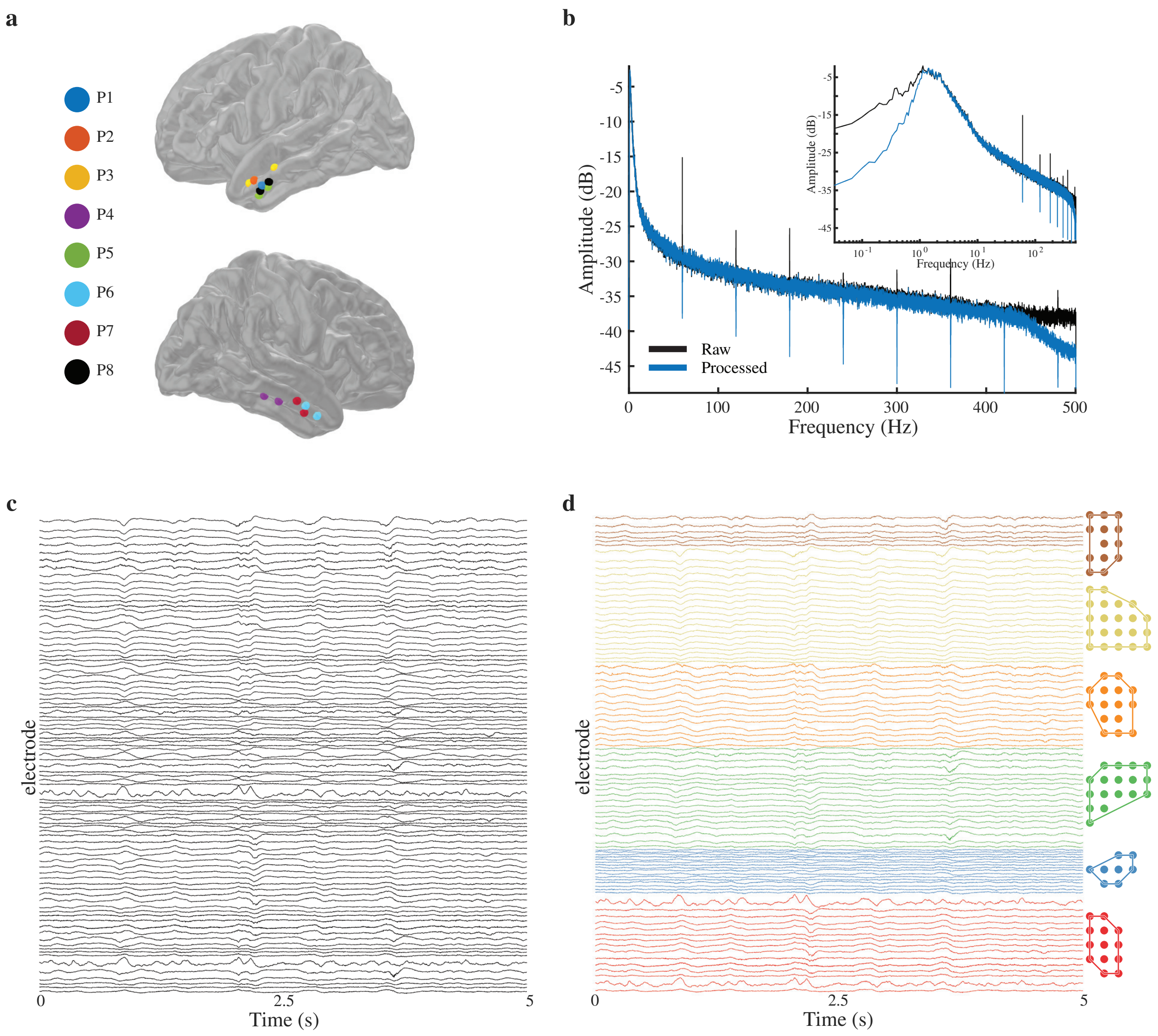

Supplementary Figure 1: **Location of implants for all MEAs and properties of recorded LFPs.**
a) Location of MEA implantation on the temporal lobe for all MEAs colored by individual participant. **b)** The main effects of the pre-processing described in methods are the removal of activity at the power line frequency (60Hz) and harmonics, and the reduction of drift and slow fluctuations below $\approx$ 1.5Hz (*inset*). There is also a small decrease in high-frequency power above $\approx$ 250Hz due to the removal of sharp artifacts. **c)** Example of a single 5-second recording block after pre-processing. **d)** Same recording block reordered and colored by module membership.

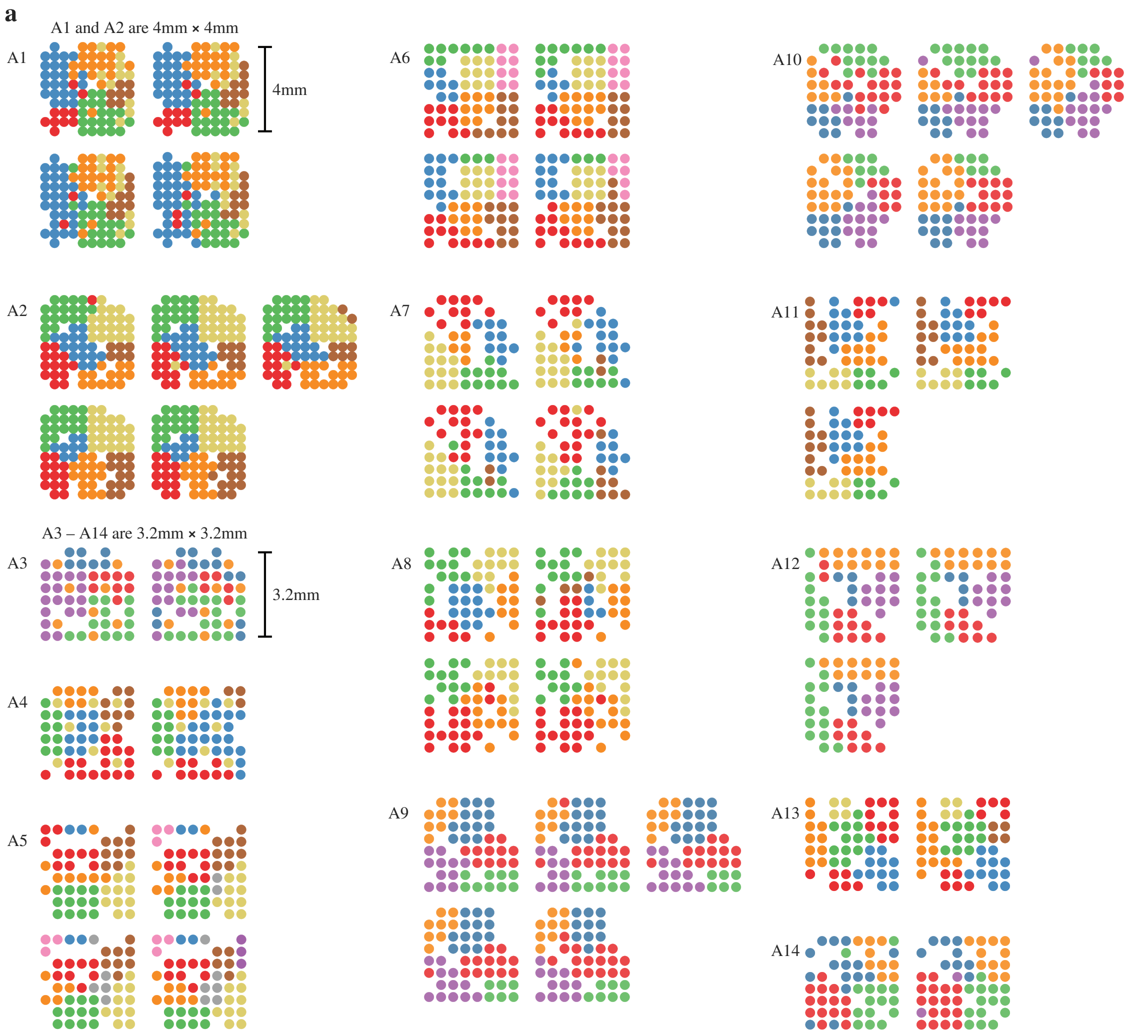

Supplementary Figure 2: **Multilayer partitions for all MEAs.**
a) For each MEA and session, each electrode is represented by a node and colored according to the multilayer partition it was assigned to. Arrays A1 and A2 are 4mm × 4mm with 96 electrodes, and arrays A3-A14 are 3.2mm × 3.2mm with 64 electrodes. Missing nodes correspond to electrodes that were rejected based on signal quality (see Methods).

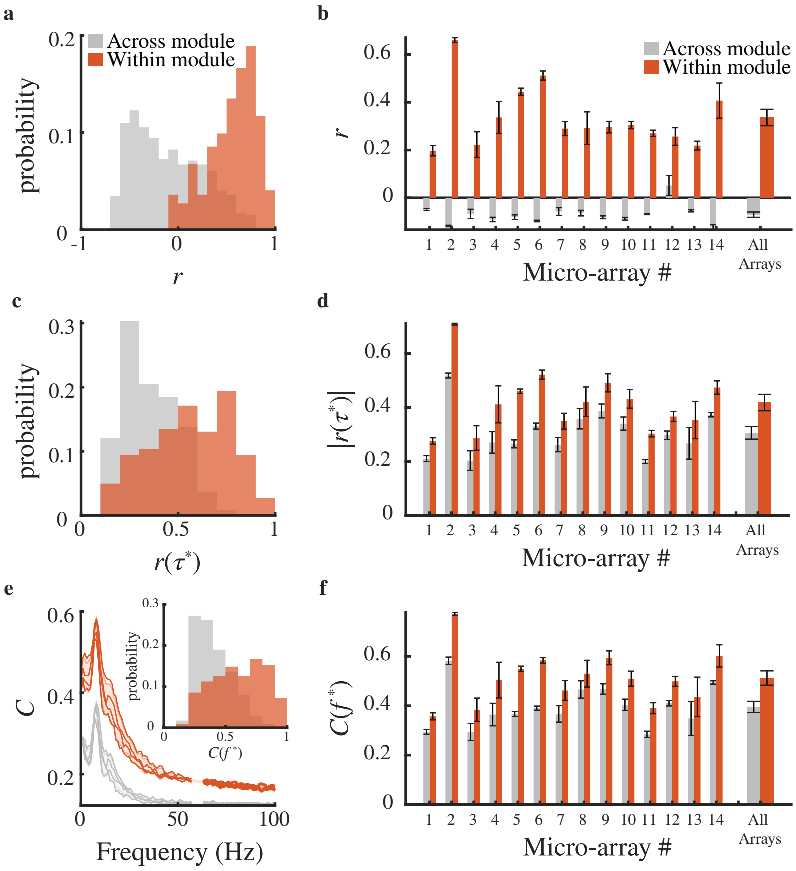

Supplementary Figure 3: **Modularity with respect to other functional connectivity metrics**.
a) Correlations in LFP activity for within vs across module pairs for a single session from an individual MEA using the module partitions derived from the GC networks. **b)** Summary for all MEAs. **c)** Same as panel **a** but using the correlogram estimate of the absolute maximum cross-correlation. **d)** Summary for all MEAs. **e)** Coherence spectrum and maximum coherence (*inset*) for within vs across module pairs for a single session and MEA using the module partitions derived from the GC networks. **f)** Summary of maximum coherence for all MEAs.

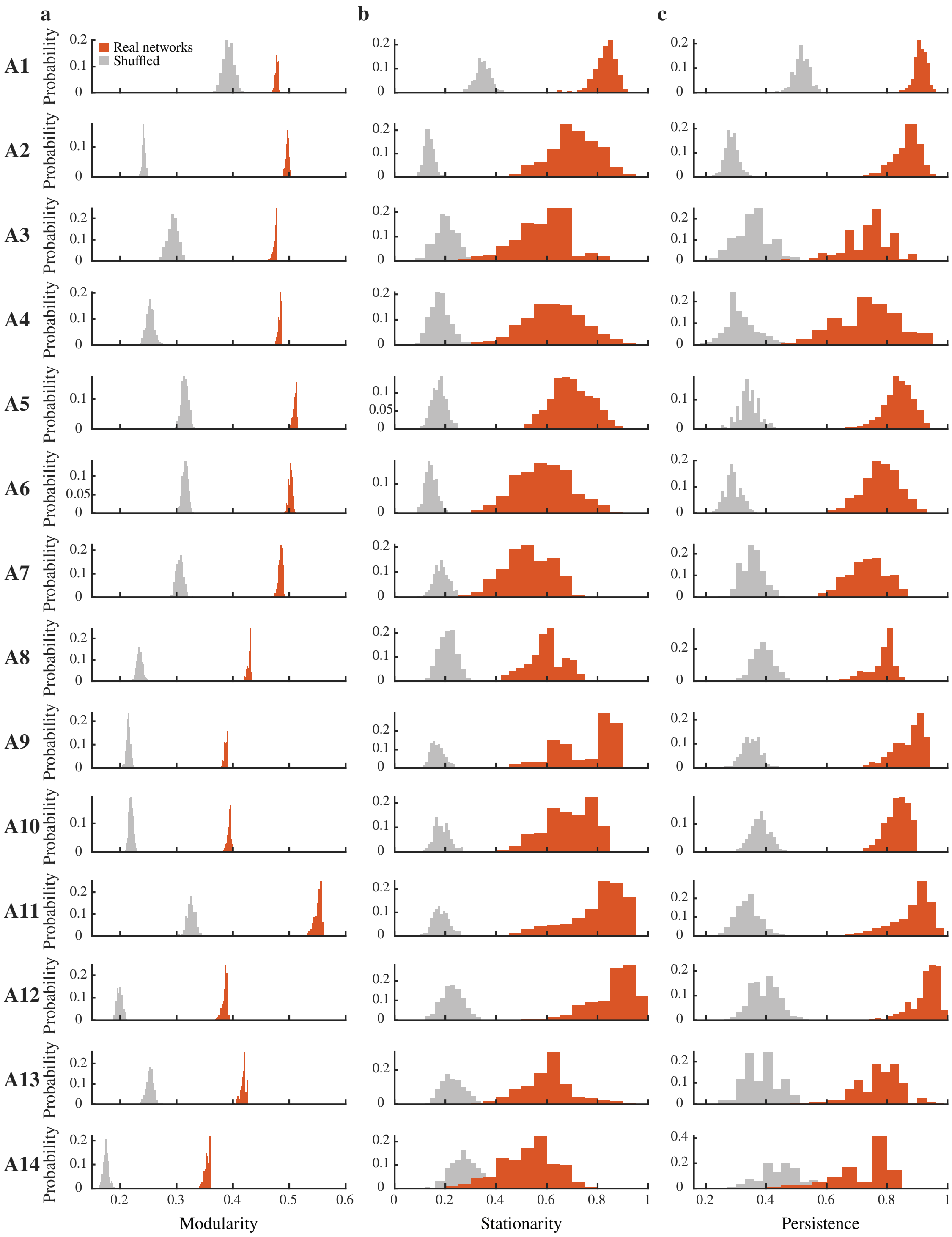

Supplementary Figure 4: **Modularity, stationarity, and persistence for all MEAs.**
a) Distribution of maximum modularity, Q_{ml}^{max}, for the $n = 500$ runs of the generalized Louvain algorithm for every MEA (orange bars). We also compute the modularity for each of the surrogate networks (gray bars). **b)** Distribution of stationarity values for the 500 runs of the generalized Louvain algorithm for real and surrogate networks. **c)** Distribution of persistence values for the 500 runs of the generalized Louvain algorithm for real and surrogate networks.

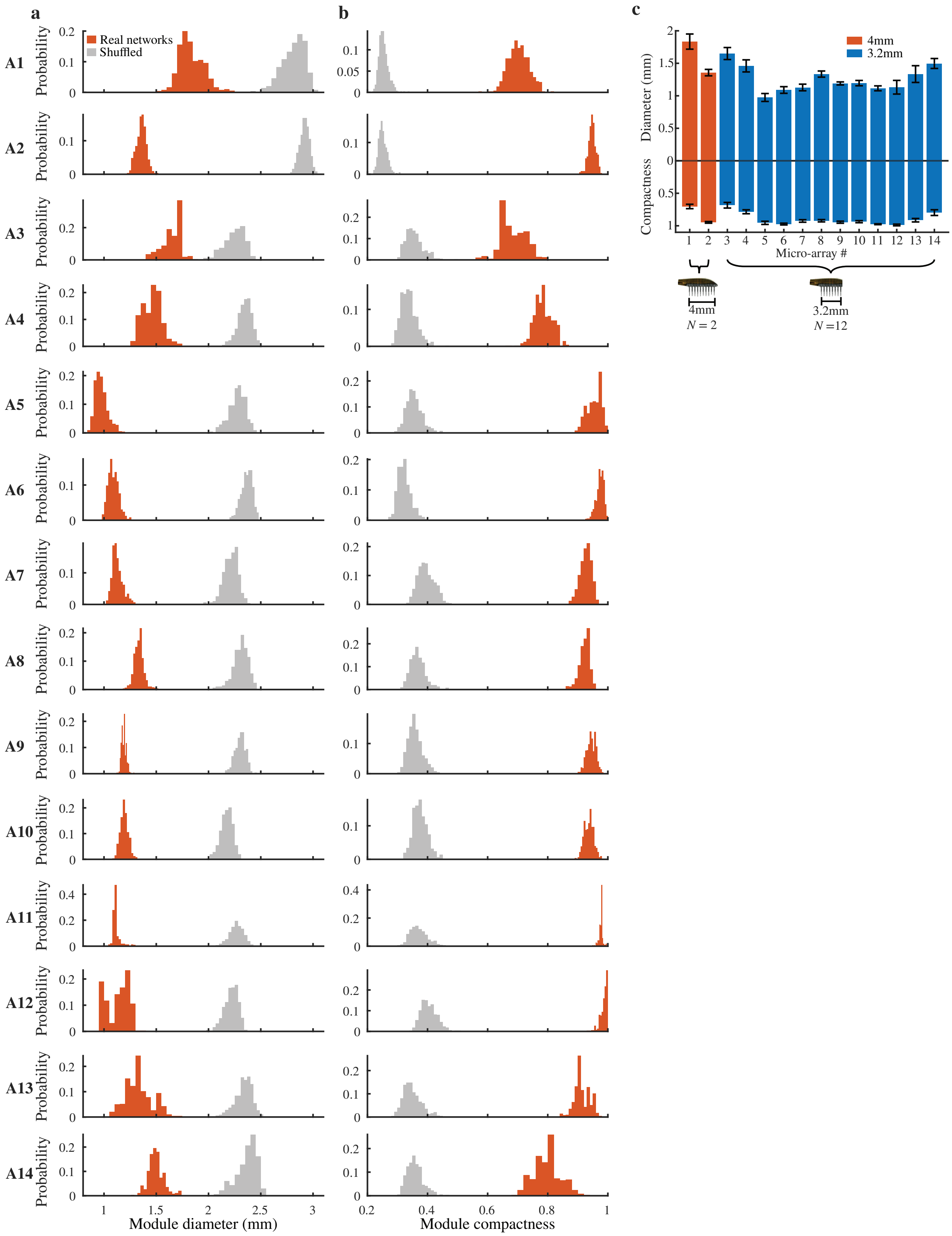

Supplementary Figure 5: **Spatial extent and module compactness for all MEAs.**
a) Distribution of module diameter for the $n = 500$ runs of the generalized Louvain algorithm for real and surrogate networks. **b)** Distribution of module compactness for the 500 runs of the generalized Louvain algorithm for real and surrogate networks. **c)** Module diameter and compactness for the 4mm $\times$ 4mm vs the 3.2mm $\times$ 3.2mm arrays.

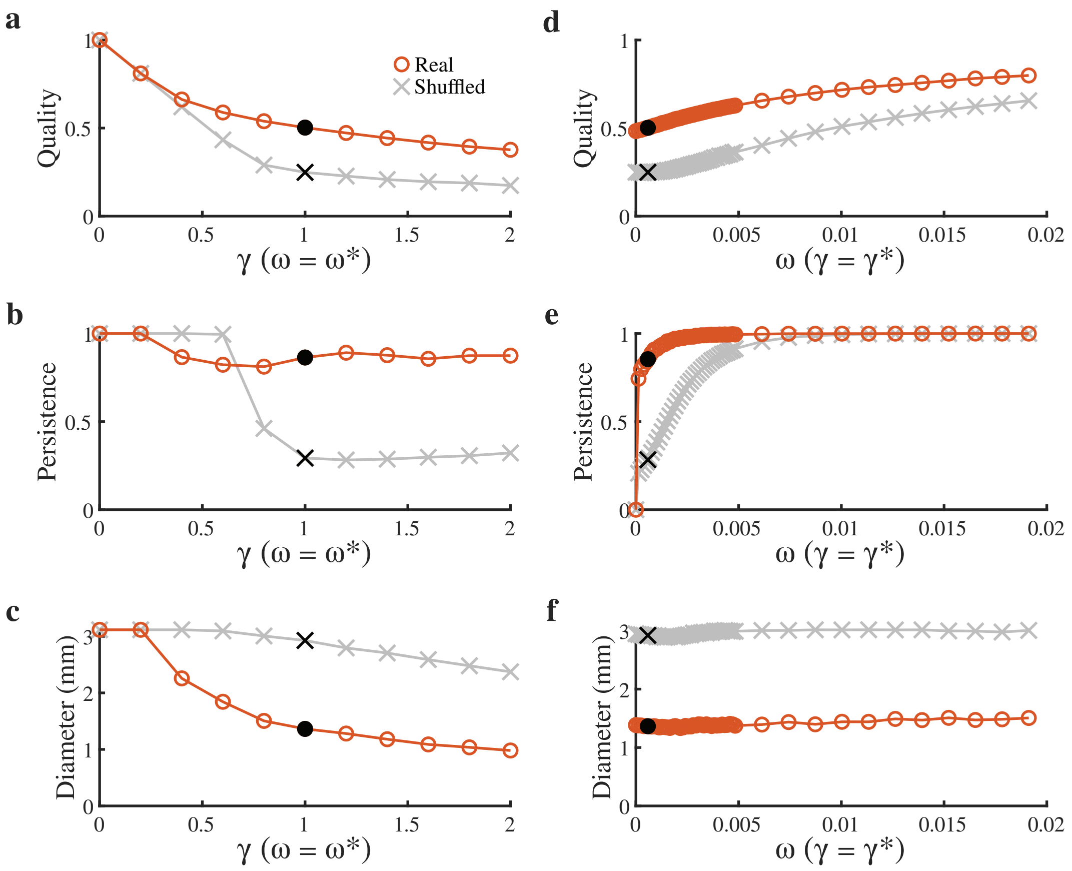

Supplementary Figure 6: **Quality, persistence, and module diameter as functions of the resolution parameter, γ, and the between-layer coupling parameter, ω. (γ^*, ω^*) denote the parameters used in the main results and are shown in black.**

a) Quality as a function of γ for a fixed $\omega = \omega^*$. **b)** Persistence as a function of γ for a fixed $\omega = \omega^*$. **c)** Module diameter as a function of γ for a fixed $\omega = \omega^*$. **d)** Quality as a function of ω for a fixed $\gamma = \gamma^*$. **e)** Persistence as a function of ω for a fixed $\gamma = \gamma^*$. **f)** Module diameter as a function of ω for a fixed $\gamma = \gamma^*$.

a

Participant ID	Microelectrode array #	# of sessions for connectivity analysis	# of image categorization sessions
P1	A1	4	0
P2	A2	5	0
P3	A3 and A4	2	0
P4	A5 and A6	4	3
P5	A7 and A8	4	2
P6	A9 and A10	5	2
P7	A11 and A12	3	1
P8	A13 and A14	2	2

b

c

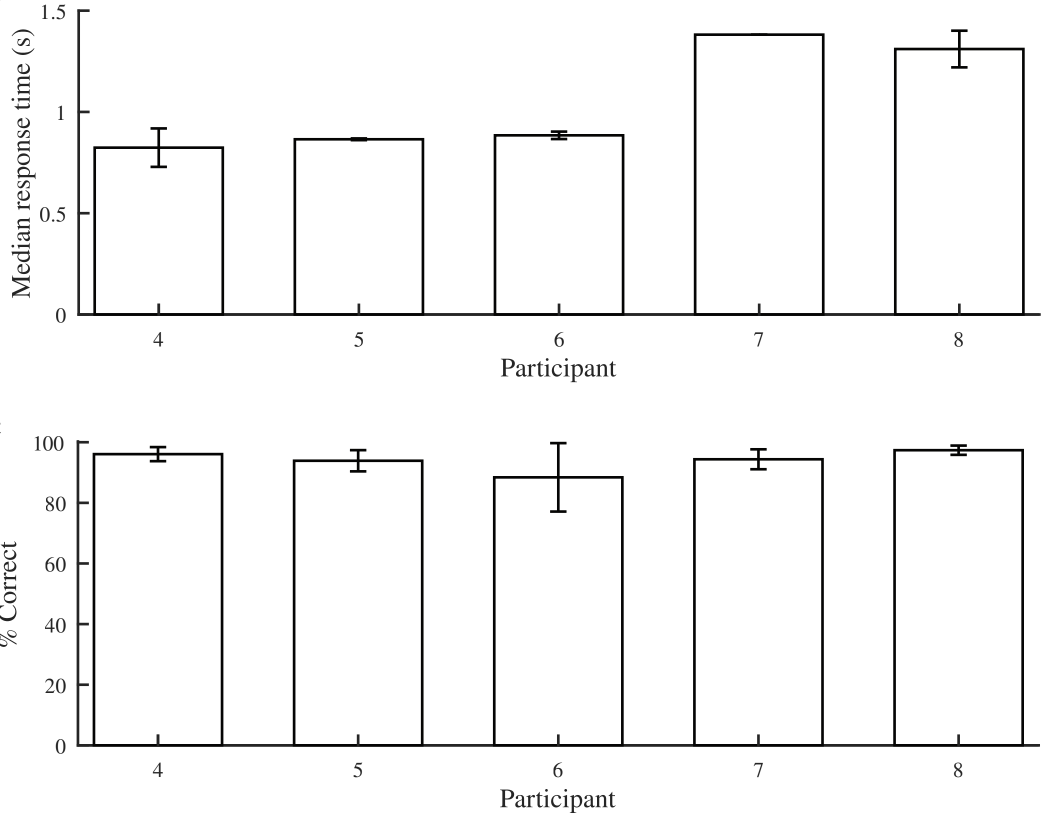

Supplementary Figure 7: **Summary of participants, arrays, sessions, and behavior during the image categorization task.**

a) Table summarizing the number of participants, microelectrode arrays, and number of sessions. **b)** Median response time. Each bar is the median response time averaged over all sessions and image categories for each participant. Error bars represent the mean and standard deviation over sessions. **c)** Percent correct. Each bar is the categorization accuracy averaged over all sessions and image categories for each participant.

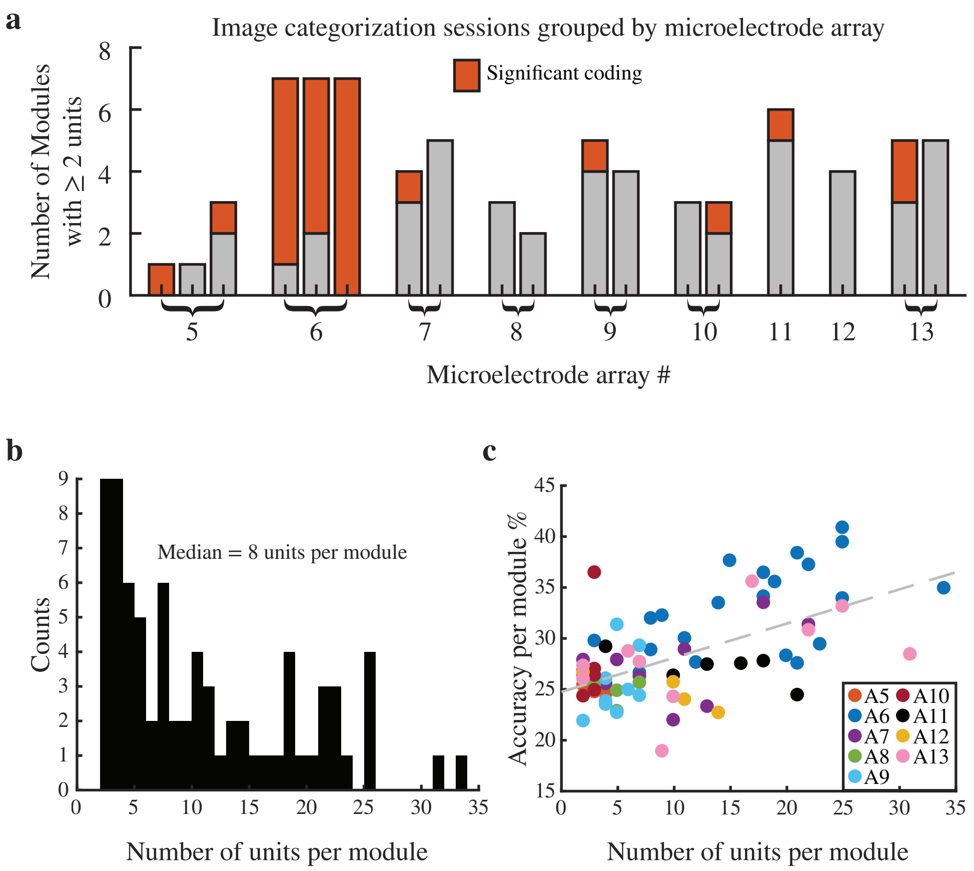

Supplementary Figure 8: **Summary of number of modules, modules with significant classification, and units per module for the image categorization task**.

a) The stacked bars represent the total number of modules for that array and session. Orange denotes modules with significant classification of image category. The sessions are grouped by MEA. **b)** Histogram with the number of units for each MEA and session. The median number of units per module is 8. **c)** Scatter plot of single module classification accuracy vs. number of units in that module. Spearman's $\rho = .51, p < .001$.

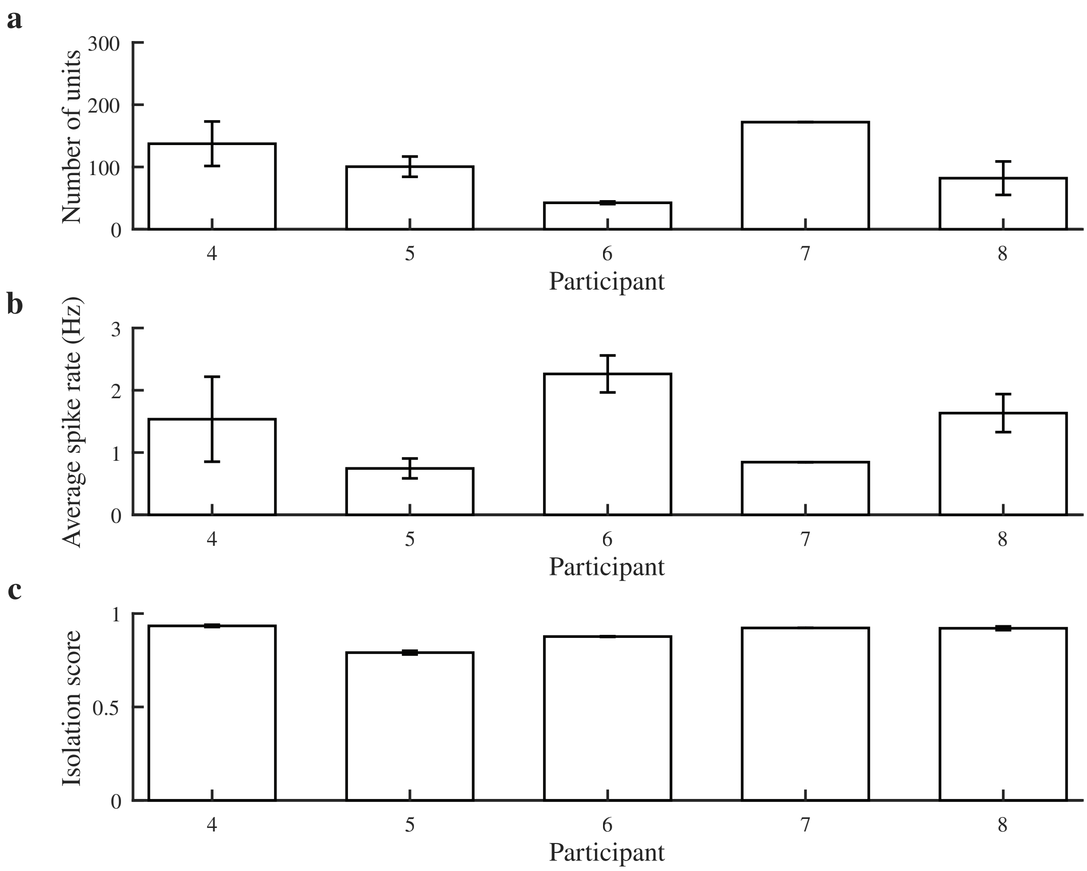

Supplementary Figure 9: **Summary of single unit metrics**.
a) Number of single units. Error bars represent the mean and standard deviation over sessions. **b)** Average spike rate. **c)** Unit isolation score.

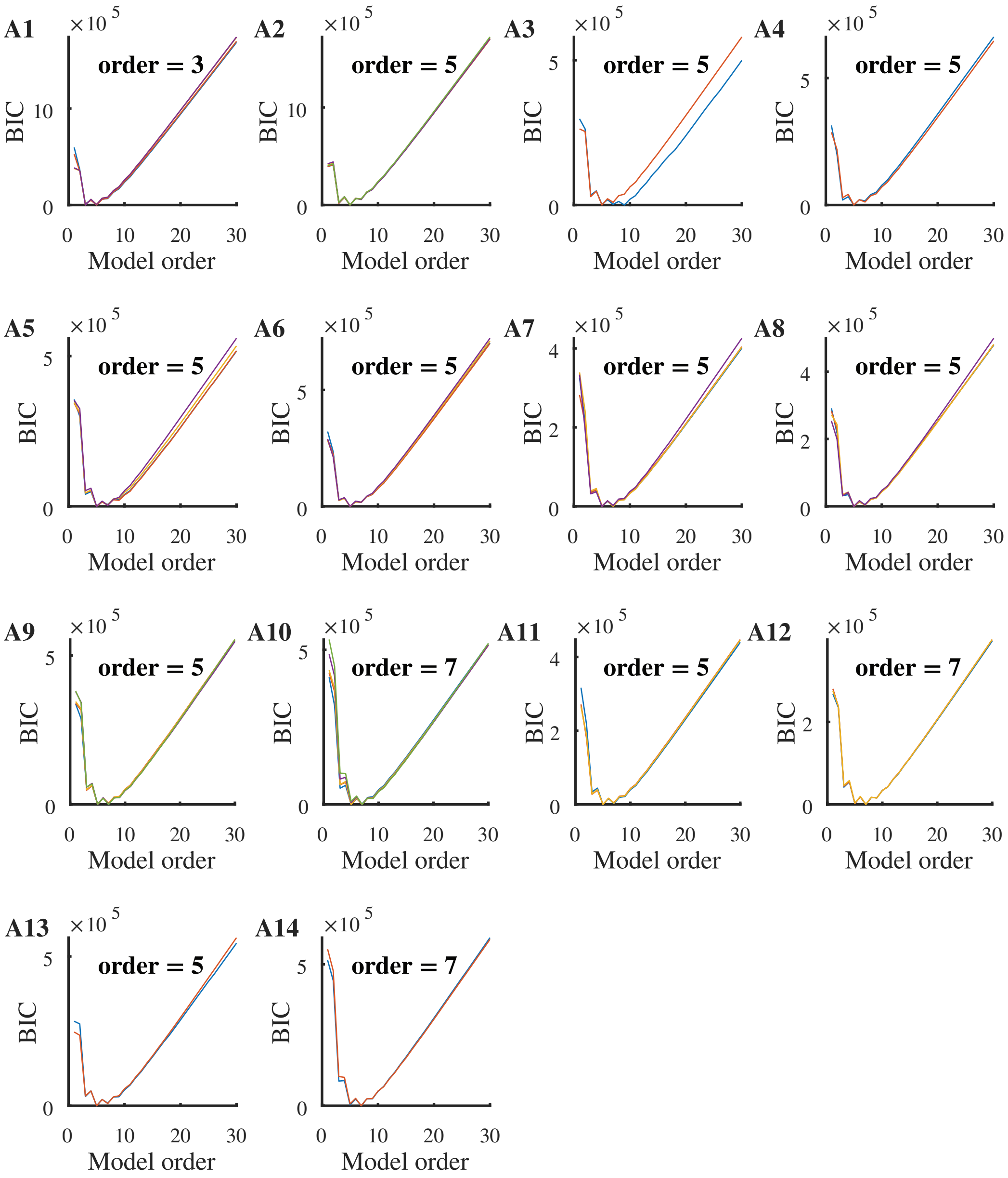

Supplementary Figure 10: **VAR model order for all MEAs**.
For each MEA and session, we compute the Bayesian Information Criterion (BIC) for the full VAR model for different model orders $1 \leq p \leq 30$. For each MEA the order for which the BIC was minimized was consistent across sessions and thus we used the same model order (the mode) for all sessions within a MEA.

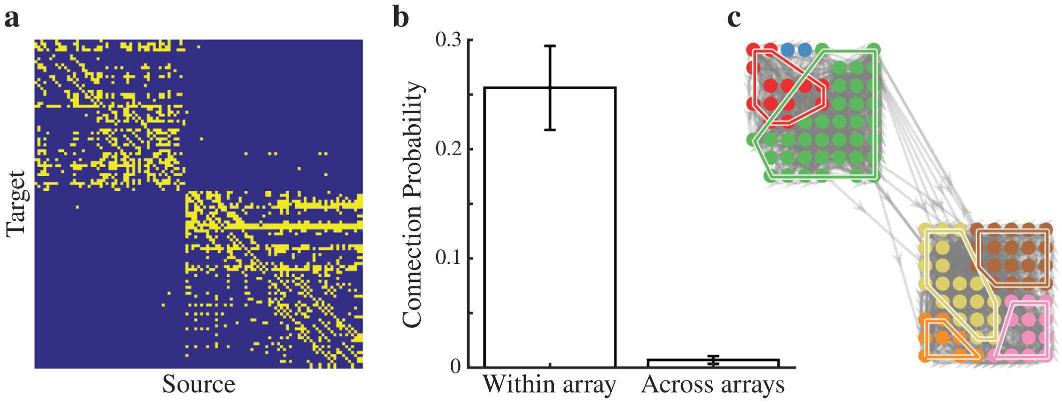

Supplementary Figure 11: **Within vs across MEA controls on connectivity and modularity**.
a) The binary adjacency matrix when computing Granger causalities using activity from 2 MEAs from a single participant shows that most of the connections are constrained within individual arrays (to left and bottom right squares). **b)** Within-array vs across-array connection probabilities for all participants with 2 MEAs. **c)** Since the activity between the two MEAs from a single participant is largely independent, modularity optimization on the combined network produces modules that are constrained to consist of electrodes from a single MEA.

Supplementary References

1. Ding, M., Chen, Y. & Bressler, S. L. Granger Causality: Basic Theory and Application to Neuroscience. In *Handbook of Time Series Analysis*, 437–460 (Wiley-VCH Verlag GmbH & Co. KGaA, Weinheim, Germany, 2006).
2. Chen, Y., Bressler, S. L. & Ding, M. Frequency decomposition of conditional Granger causality and application to multivariate neural field potential data. *Journal of Neuroscience Methods* **150**, 228–237 (2006).
3. Seth, A. K. A MATLAB toolbox for Granger causal connectivity analysis. *Journal of Neuroscience Methods* **186**, 262–273 (2010).
4. Bressler, S. L. & Seth, A. K. Wiener-Granger Causality: A well established methodology. *NeuroImage* **58**, 323–329 (2011).
5. Barnett, L. & Seth, A. K. The MVGC multivariate Granger causality toolbox: A new approach to Granger-causal inference. *Journal of Neuroscience Methods* **223**, 50–68 (2014).
6. Bastos, A. M., Vezoli, J. & Fries, P. Communication through coherence with inter-areal delays. *Current Opinion in Neurobiology* **31**, 173–180 (2015).
7. Michalareas, G. *et al.* Alpha-Beta and Gamma Rhythms Subserve Feedback and Feedforward Influences among Human Visual Cortical Areas. *Neuron* **89**, 384–397 (2016).
8. Vezoli, J. *et al.* Brain rhythms define distinct interaction networks with differential dependence on anatomy. *Neuron* **109**, 3862–3878.e5 (2021).
