# Supplementary material for: Micro-scale functional modules in the human temporal lobe
Source: Nat Commun. 2022 Oct 21;13:6263. doi: 10.1038/s41467-022-34018-w (PMC9587217; doi:10.1038/s41467-022-34018-w)
Supplement: Supplementary file 3 — Reporting Summary [file 41467_2022_34018_MOESM3_ESM.pdf]

nature portfolio

Corresponding author(s): Kareem A. Zaghloul

Last updated by author(s): Sep 7, 2022

Reporting Summary

Nature Portfolio wishes to improve the reproducibility of the work that we publish. This form provides structure for consistency and transparency in reporting. For further information on Nature Portfolio policies, see our Editorial Policies and the Editorial Policy Checklist.

Statistics

For all statistical analyses, confirm that the following items are present in the figure legend, table legend, main text, or Methods section.

n/a	Confirmed	
☐	☒	The exact sample size (*n*) for each experimental group/condition, given as a discrete number and unit of measurement
☐	☒	A statement on whether measurements were taken from distinct samples or whether the same sample was measured repeatedly
☐	☒	The statistical test(s) used AND whether they are one- or two-sided<br>*Only common tests should be described solely by name; describe more complex techniques in the Methods section.*
☐	☒	A description of all covariates tested
☐	☒	A description of any assumptions or corrections, such as tests of normality and adjustment for multiple comparisons
☐	☒	A full description of the statistical parameters including central tendency (e.g. means) or other basic estimates (e.g. regression coefficient) AND variation (e.g. standard deviation) or associated estimates of uncertainty (e.g. confidence intervals)
☐	☒	For null hypothesis testing, the test statistic (e.g. *F*, *t*, *r*) with confidence intervals, effect sizes, degrees of freedom and *P* value noted<br>*Give P values as exact values whenever suitable.*
☒	☐	For Bayesian analysis, information on the choice of priors and Markov chain Monte Carlo settings
☒	☐	For hierarchical and complex designs, identification of the appropriate level for tests and full reporting of outcomes
☒	☐	Estimates of effect sizes (e.g. Cohen's *d*, Pearson's *r*), indicating how they were calculated

Our web collection on statistics for biologists contains articles on many of the points above.

Software and code

Policy information about availability of computer code

Data collection	Microelectrode signals were digitally recorded at 30 kHZ using the Cereplex I and a Cerebus acquisition (Blackrock Microsystems, Salt Lake City, UT).
Data analysis	Single units were isolated using Plexon Offline Sorter version 4.0. The chronux_2_11 toolbox was used for signal preprocessing, the mvgc_v1.0 toolbox was used for all Granger causal calculations, and the SHINE toolbox was used to balance image properties. All remaining analyses were performed using custom code in MATLAB R2020b (Mathworks, Inc.). The custom MATLAB scripts used for analysis are available upon request.

For manuscripts utilizing custom algorithms or software that are central to the research but not yet described in published literature, software must be made available to editors and reviewers. We strongly encourage code deposition in a community repository (e.g. GitHub). See the Nature Portfolio guidelines for submitting code & software for further information.

Data

Policy information about availability of data

All manuscripts must include a data availability statement. This statement should provide the following information, where applicable:

- Accession codes, unique identifiers, or web links for publicly available datasets
- A description of any restrictions on data availability
- For clinical datasets or third party data, please ensure that the statement adheres to our policy

The data that support the findings of this study are available at https://research.ninds.nih.gov/zaghloul-lab/downloads and also from the corresponding author upon request.

Field-specific reporting

Please select the one below that is the best fit for your research. If you are not sure, read the appropriate sections before making your selection.

☒ Life sciences ☐ Behavioural & social sciences ☐ Ecological, evolutionary & environmental sciences

For a reference copy of the document with all sections, see nature.com/documents/nr-reporting-summary-flat.pdf

Life sciences study design

All studies must disclose on these points even when the disclosure is negative.

Sample size	No sample-size calculations were carried out. We used all available participants that met our inclusion criteria at the start of this study.
Data exclusions	We included participants that had at least 2 experimental sessions with a minimum duration of 30 minutes each. Any sessions with consistent artifacts, electrical noise, or during which electrical stimulation was being applied were excluded. This resulted in 8 participants.
Replication	We did not replicate these results in a separate cohort of participants. The data presented here were captured over several years from microelectrode array recordings in human neurosurgical patients receiving treatment for epilepsy, and are thus extremely rare. However, we did require that each participant had at least 2 experimental sessions for connectivity analyses, and confirmed that the modules we identified are reproducible from day to day.
Randomization	Randomization of participants was not relevant to this study as there was no group allocation during data collection or analysis.
Blinding	Blinding was not relevant to this study as there was no group allocation during data collection or analysis.

Reporting for specific materials, systems and methods

We require information from authors about some types of materials, experimental systems and methods used in many studies. Here, indicate whether each material, system or method listed is relevant to your study. If you are not sure if a list item applies to your research, read the appropriate section before selecting a response.

Materials & experimental systems

n/a	Involved in the study	
☒	☐	Antibodies
☒	☐	Eukaryotic cell lines
☒	☐	Palaeontology and archaeology
☒	☐	Animals and other organisms
☐	☒	Human research participants
☒	☐	Clinical data
☒	☐	Dual use research of concern

Methods

n/a	Involved in the study	
☒	☐	ChIP-seq
☒	☐	Flow cytometry
☒	☐	MRI-based neuroimaging

Human research participants

Policy information about studies involving human research participants

Population characteristics	Eight participants (4 female; 39.3 ± 9.5 years old; mean ± SD) with implanted microelectrode arrays.
Recruitment	Participants were recruited on the basis of clinical need for surgical epilepsy localization. There were no patients that were selected or excluded based on patient characteristics.
Ethics oversight	The Institutional Review Board of the National Institutes of Health and the National Institute of Neurological Disorders and Stroke approved the experimental protocol (11-N-0051 Epilepsy Surgery) through which these data were collected.

Note that full information on the approval of the study protocol must also be provided in the manuscript.
